# Brain histamine and oleoylethanolamide restore behavioral deficits induced by chronic social defeat stress in mice

**DOI:** 10.1016/j.ynstr.2021.100317

**Published:** 2021-03-17

**Authors:** Barbara Rani, Andrea Santangelo, Adele Romano, Justyna Barbara Koczwara, Marzia Friuli, Gustavo Provensi, Patrizio Blandina, Maurizio Casarrubea, Silvana Gaetani, Maria Beatrice Passani, Alessia Costa

**Affiliations:** a Dipartimento di Scienze della Salute, Università di Firenze (I), Italy; b Dipartimento di Fisiologia e Farmacologia ‘V. Erspamer’, Sapienza Università di Roma, Roma, Italy; c Dipartimento di Neuroscienze, Psicologia, Area del Farmaco e Salute del bambino (Neurofarba) Università di Firenze Viale Pieraccini 6, 50139, Firenze Italy; d Dipartimento di Biomedicina, Neuroscienze e Diagnostica Avanzata (Bi.N.D.), Sezione di Fisiologia Umana “Giuseppe Pagano”, Università degli Studi di Palermo, Corso Tukory 129, 90134, Palermo, Italy

**Keywords:** T-pattern analysis, Recognition memory, Oxytocin, Social interaction, Histidine decarboxylase

## Abstract

The physiological mechanisms underlying the complex interplay between life stressors and metabolic factors is receiving growing interest and is being analyzed as one of the many factors contributing to depressive illness. The brain histaminergic system modulates neuronal activity extensively and we demonstrated that its integrity is necessary for peripheral signals such as the bioactive lipid mediator oleoylethanolamide (OEA) to exert its central actions. Here, we investigated the role of brain histamine and its interaction with OEA in response to chronic social defeat stress (CSDS), a preclinical protocol widely used to study physio-pathological mechanisms underlying symptoms observed in depression. Both histidine decarboxylase null (HDC^−/-^) and HDC^+/+^ mice were subjected to CSDS for 21 days and treated with either OEA or vehicle daily, starting 10 days after CSDS initiation, until sacrifice. Undisturbed mice served as controls. To test the hypothesis of a histamine-OEA interplay on behavioral responses affected by chronic stress, tests encompassing the social, ethological and memory domains were used. CSDS caused cognitive and social behavior impairments in both genotypes, however, only stressed HDC^+/+^ mice responded to the beneficial effects of OEA. To detect subtle behavioral features, an advanced multivariate approach known as T-pattern analysis was used. It revealed unexpected differences of the organization of behavioral sequences during mice social interaction between the two genotypes. These data confirm the centrality of the neurotransmitter histamine as a modulator of complex behavioral responses and directly implicate OEA as a protective agent against social stress consequences in a histamine dependent fashion.

## Highlights

• Chronic social defeat stress causes behavioural deficits in mice.
• Subchronic administration of oleoylethanolamide prevents behavioural impairment of stressed wild type mice.
• Oleoylethanolamide is ineffective in stressed mice lacking brain histamine (HDC^−/-^ mice).
• T-pattern analysis several differences in the impact of stress and oleoylethanolamide in the two genotypes.

## 1 Introduction

Histamine and OEA are phylogenetically old molecules that have been described in several species, from Drosophila (Tortoriello et al., 2013) (Denno et al., 2016) to humans (Schaefer et al., 2014) (Panula and Nuutinen, 2013). They interact directly or indirectly to control metabolic (Misto et al., 2019) as well as behavioural responses (Costa et al., 2018; Provensi et al., 2017). Presumably via vagal stimulation and a multisynaptic pathway, OEA increases the activity of a small population of histaminergic neurons in the hypothalamic tuberomamillary nucleus (TMN) thus augmenting histamine release in the cortex of fasted mice (Provensi et al., 2014).

Our recent studies demonstrate that all central actions of OEA investigated, invariably necessitate the activation of the brain histaminergic system. For instance, depletion of brain histamine blunts OEA-induced hypophagia in mice (Provensi et al., 2014). Also, the histaminergic neurotransmission serves as a gateway for OEA to exert cognitive effects in contextual fear memory (Provensi et al., 2017) and antidepressant-like effects in an acute stress based model, the tail suspension test (Benetti et al., 2015; Costa et al., 2018; Provensi et al., 2014). Despite being a valuable tool in drug discovery for high-throughput screening of prospective antidepressants, the tail suspension test is inadequate to investigate the neurobiological substrates of chronic stress and the pathogenesis of mood disorders. There are several models of chronic stress and anxiety, ranging from milder protocols, to more sever ones, such as maternal deprivation that mimics stress experienced in early life, or chronic social defeat (Miczek, 1979**)** that more closely represents exposure to stress stimuli in adulthood humans. Indications of the potential protective effect of OEA against chronic unpredictable stress-induced metabolic and behavioural impairments were described by (Bortolato et al., 2007) and (Jin et al., 2015), respectively. Moreover, recent evidence suggests that OEA decreases frustration stress-induced binge-like eating in female rats (Romano et al., 2020), thus further supporting the potential beneficial effect of OEA in stress-conditions.

There is extensive evidence that histaminergic neurons detect acute stress-induced signals. Exposure to restraint and cold increased histamine turn over in the rat hypothalamus (Taylor and Snyder, 1971); hypercapnic loading (Haxhiu et al., 2001), insulin-induced hypoglycaemia and foot shock (Haxhiu et al., 2001; Miklós and Kovács, 2003) activated histaminergic neurons in a stressor- and neuron subgroup-specific manner. Furthermore, increased histamine release was observed in the cortex of freely moving rats during handling (Westerink et al., 2002) and in the TMN of hungry rats enticed with food enclosed in a wire mesh (Valdés et al., 2010). In this framework it is not surprising that the histaminergic circuits modulate the formation and retrieval of memories associated with aversive events (Benetti et al., 2015; Fabbri et al., 2016).

Here, we explored the relationship between the histaminergic system and OEA on the behavioral outcomes of chronic social defeat stress, a preclinical paradigm that more closely reproduces some of the symptoms observed in depression (Menard et al., 2017). To address our questions, we used genetically modified mice that do not express histidine decarboxylase, the only enzyme responsible for histamine synthesis (HDC^−/-^ mice) and wild type mice (HDC^+/+^) which were subjected to a 21-day social defeat protocol and administered OEA starting 10 days after the beginning of the stress. We then used a battery of tests comprehensive of several domains potentially affected by chronic stress such as social interaction, behavioral sequences complexity and short-term memory.

The peptide oxytocin is a regulator of anxiety, stress, coping and sociability (reviewed in (Neumann and Landgraf, 2012). OEA stimulates oxytocin neurosecretion from the paraventricular nucleus (PVN) of the hypothalamus and enhances oxytocin expression at both axonal and somatodendritic levels of hypothalamic neurons (Romano et al., 2013). The physiological role of these effects have been extensively demonstrated as OEA releases oxytocin to induce satiety (Gaetani et al., 2010) and we previously demonstrated that this occurs in a histamine-dependent way (Provensi et al., 2014; Umehara et al., 2016).

Based on these aforementioned observations, in the present study, we also investigated the capability of peripheral administered OEA to modulate oxytocin immunoreactivity in the PVN of stressed and non-stressed HDC^+/+^ and HDC^−/-^ mice.

Taken together, our results strongly suggest that OEA ameliorates stress-related behaviours in a histamine dependent manner, providing further support to our general hypothesis that the histaminergic system allocates to peripheral stimuli (in this case OEA) the salience necessary to unfold the appropriate behaviours.

## 2 Material and methods

### 2.1 Animals

**Fig. 1:**
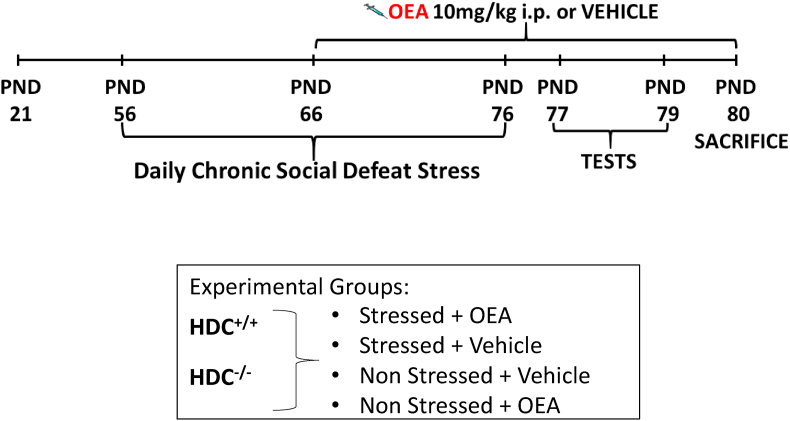
Timeline for the chronic social defeat stress protocol and OEA or vehicle injections. Mice were randomly assigned to **four** experimental groups per genotype.

Histidine decarboxylase null (C57bl/6, HDC^−/-^) and wild type (C57bl/6, HDC^+/+^) mice were grown in the Centro Stabulazione Animali di Laboratorio (CeSAL), Università di Firenze in humidity, temperature (22–24 °C) and light (light on 7:00-19:00)-controlled room. Mice were allowed free access to food and water. At postnatal (PND) day 21, mice were weaned and fed with standard chow diet (Mucedola s.r.l., Milan, Italy). Nine to 13-week-old male CD1 retired breeders (Charles River, Italy) were screened for aggressive behavior and used for the social defeat stress protocol according to (Golden et al., 2011). All experiments were performed in accordance with the EEC recommendations for the care and use of laboratory animals (2010/63/EU) and approved by the Animal Care Committee of the University of Florence and Italian Ministry of Health (authorization n. 114-2017 PR) and complying to the 3R. Ethical policy of the Universitá di Firenze complies with the Guide for the Care and Use of Laboratory Animals of the Council Directive of the European Community (2010/63/EU) and the Italian Decreto Legislativo 26 (13/03/2014). Every effort was made to minimize animal suffering and to reduce the number of animals used. All animals were weighted, and food consumption calculated daily. OEA (Tocris Bioscience, UK) was administered (10 mg/kg) by i.p. injections in a solution of saline/polyethylene glycol/Tween 80 (90/5/5, v/v/v). Control, non-stressed mice received the injection of OEA or vehicle (VEH). Both vehicle and OEA solutions were freshly prepared on each test day and administered during the light-phase of the animals, in the morning between 9:00 and 10:30 a.m. from PND 66 until sacrifice (Fig. 1).

### 2.2 Chronic social defeat stress

C57bl/6 mice were singly housed prior to undergoing social defeat stress. CD1 mice were used as resident aggressors for the social defeat stress and were singly-housed prior to the experiments. Aggressive CD-1 mice, as defined by demonstrating at least one successful act of aggression during two consecutive days toward another male CD1 intruder mouse, were selected for use in the social defeat. A group of HDC^+/+^ and of HDC^−/-^ mice that received vehicle or OEA of either genotype were subjected to the CSDS protocol for 21 days (PND56 to PND76) according to (Bartolomucci et al., 2001; Finger et al., 2012; Reber et al., 2006) with minor modifications. Specifically, the timing of stress procedure was chosen based on the experimental design. Here we used a compound, OEA, with known antidepressant-like effects (Costa et al., 2018), that was administered daily immediately before the social defeat encounters or the overcrowding sessions. The therapeutic effects of antidepressant compounds require 1–2 weeks to first appear. Accordingly, we decided to start the pharmacological intervention after 10 days of CSDS, when stress-induced alterations are manifested, and not beforehand (see for review (Pryce and Fuchs, 2017). The OEA treatment was followed across a relevant time period, as previously described (Di Paola et al., 2018), and given daily throughout the stressful procedure and behavioural assessment. Briefly, the CSDS procedure consisted of the introduction of an experimental mouse of either genotype in the cage of a CD-1 aggressor until the first aggression occurred. Mice were then separated for 2 h by a transparent, perforated Plexiglas wall to allow visual and olfactory exposure. The separator was then removed, and the second attack occurred. Social defeat sessions were carried out once daily (on days 1–4, 7–10, 15, 16, 19–21) or twice daily (on days 6, 12 and 17). The stress protocol included overcrowding sessions: 6/8 mice were placed together in a standard holding cage (33 × 15 × 13 cm) for 24 h (on days 5–6, 11–12, 18–19) or 48 h (on days 13–15) with diet and water available *ad libitum.* Non-stressed mice were left undisturbed in their own home cages with other non-stressed mice (4 mice per cage).

### 2.3 Open field test

Mice locomotor activity and anxiety-like level were tested in an open arena (60 x 70 × 30 cm) where a virtual zone (20 × 23 cm) was delimited in the center of the arena; mice were allowed to freely explore the arena for 10 min. In between observations, the arena was cleaned with 30% ethyl alcohol in water to remove possible scent cues left by the animal. The time spent at the center and periphery of the open field and total distance travelled were measured using a video tracking system and analyzed using Smart 2.5 software.

### 2.4 Social interaction test

Twentyfour hours after the last defeat session mice were subjected to the social interaction test adopting the protocol by (Golden et al., 2011). Briefly, mice were habituated to an arena (41 cm × 32 cm x 40 cm) containing an empty wire-mesh enclosure (7.5 cm length, 9.5 cm width) and their movements recorded for 2.5 min to determine baseline exploratory behavior and locomotion (T1). During the second session (T2) the wire-mesh contained an unfamiliar CD1 aggressive mouse and the time the experimental C57BL/6 mouse spent in its proximity was measured. Trials were video-recorded and analyzed by an experienced observer unaware of the group assignment to time spent in the interaction zone, that is 5 cm around the wire mesh cage. Exploration was defined as sniffing or touching the cage with the nose and/or forepaws. Social interaction (SI) was calculated as the ratio between the time spent in the interaction zone during T2 and T1.

### 2.5 Ethological assessment: experimental procedure, quantitative and T-pattern analyses

The procedure of the ethological analysis was carried out as described in (Santangelo et al., 2017). The ethogram (namely, a formal list of each component of the behavioral repertoire and its description) encompassing *Cage related* and *Other behaviors* is shown in Table 1. On the basis of this ethogram, each video file was analyzed by means of The Observer software tool (Noldus Information Technology bv, The Netherlands) and event log files (i.e., the behaviors performed and their onset) was obtained. Event log files were used to perform quantitative evaluations (Occurrences and Durations of each behavioral component) and T-pattern analysis (TPA). Measurements of occurrence and duration of each component of the behavioral repertoire were based on the descriptive analysis shown in Table 2. The TPA is a multivariate technique able to detect the existence of statistically significant constraints among behavioral events in the course of time. TPA orbits around the utilization of a software tool known as Theme (PatternVision, ltd, Iceland) and the result of such a detection procedure are the T-patterns. Simply stated, a T-pattern represents the detection of repetitive features of a behavior (Casarrubea et al., 2015, 2018) (Magnusson, 2020) and can be summarized using the following notation:

X1 ≈dt1 X2 ≈dt2 X3 ... Xi ≈ dti Xi+1 ... Xm−1 ≈dtm−1 Xm

where X1 ... X2 ... Xm terms highlight the events of the given T-pattern and ≈dt indicates the temporal distances separating these events. For instance, the term Xi ≈ dti Xi+1 indicates that the Xi event is followed by time units (≥0) later by the following Xi+1 event. Basically, a detection algorithm operating within a given time window encompassing a number of events (e.g., A, B, C, D …) compares the distribution of each pair of events (e.g. A and B) searching for a time interval so that the event A is followed by the event B, within that interval, more often than expected by mere chance. If such a circumstance, statistically verified, exists, A and B are by definition a T-pattern and are indicated as (A B). In a second step, such a first level T-pattern (A B) is considered as potential “A” or “B” term to form higher order patterns, e.g. ((A B) C), ((A B)(C D), and so on. Such a bottom-up detection process continues up to any level and is completed when no more patterns are found. Before a search is run, THEME software requires some parameters to be adjusted. Selected parameters were: “Critical interval type” = free; “Univariate Patterns” = include; “Significance Level” = 0.0001; “Lumping Factor” = 0.90; “Minimum % Of Samples” = 100%.

**Table 1:** Ethogram of mice behavior during the social interaction test. These comprise events performed in the proximity of the cage holding the CD1 mouse (*Cage Related Events*) and *Other Events* that occur in the arena distant from the encaged CD1 mouse.

ETHOGRAM	
CAGE RELATED EVENTS	
**Cage Sniffing - CS**	the mouse sniffs Cage borders and/or ground
**Cage Leaning - CL**	the mouse maintains an erect posture by leaning against Cage walls
**Cage Climbing - CC**	the mouse mounts on Cage walls and roof. At least three paws grab the Cage grid
**Cage Retraction - CR**	the mouse suddenly retracts its head-shoulder segment or its body far from the Cage
**NON CAGE RELATED (OTHER) EVENTS**	
**Walking - Wa**	the mouse walks in the arena. Sniffing activities may be produced if locomotion continues
**Place Sniffing - PS**	the mouse sniffs the surrounding arena environment without walking activity. Head and vibrissae movements are produced. If the mouse sniffs the central Cage borders and/or ground the correct annotation is *Cage Sniffing*
**Stretched Sniffing - SS**	the mouse stretches its head and shoulders forward and then returns to the original position. Anterior limbs stand still
**Wall Leaning - WL**	the mouse maintains an erect posture by leaning against arena walls
**Rearing - Re**	the mouse maintains an erect posture without leaning against the walls
**Jumping - Ju**	the mouse leaps from the surface of the arena
**Fore Body Grooming - FBG**	the mouse licks or rubs its face and/or its anterior limbs
**Hind Body Grooming - HBG**	tHe mouse licks or rubs its body fur and/or its posterior limbs;
**Immobility - Im**	the mouse maintains a fixed posture

**Table 2:** The table lists the occurrence and duration of each behavioral component in the total population of mice divided by genotype and treatment. See Table 1 for abbreviations.

OCCURRENCES																				
	**HDC**^**+/+**^								**HDC**^**−/−**^											
	**NS-VEH**		**S-VEH**		**NS-OEA**		**S-OEA**		**NS-VEH**			**S-VEH**		**NS-OEA**		**S-OEA**				
**Behavior**	**Mean (n)**	**SE**	**Mean**	**SE**	**Mean**	**SE**	**Mean**	**SE**	**Mean (s)**	**SE**		**Mean**	**SE**	**Mean**	**SE**	**Mean**	**SE**	F	p	
**cs**	31.00	1.95	9.20	1.93	23.00	2.13	18.00	5.32	16.00	1.57		14.67	4.14	21.17	3.74	8.67	2.79	**9.650**	**0.004**	
**cl**	5.60	1.63	0.40	0.24	6.67	0.71	1.00	0.63	0.67	0.49		0.50	0.34	5.50	1.41	0.17	0.17	–	–	
**cc**	0.80	0.37	0.00	0.00	0.67	0.49	0.00	0.00	0.00	0.00		0.00	0.00	0.83	0.65	0.00	0.00	–	–	
**cr**	3.40	2.93	4.80	0.73	2.17	0.87	6.80	1.16	8.17	0.60		9.50	2.93	2.33	1.05	6.33	1.94	0.013	0.910	
**wa**	42.40	3.04	25.60	2.98	34.50	2.93	28.20	7.74	29.50	4.42		25.50	4.19	20.67	4.12	22.00	5.16	0.163	0.689	
**ps**	27.40	1.96	26.80	1.66	26.83	1.58	30.40	3.16	31.83	2.77		28.00	2.53	24.67	3.11	26.50	3.59	0.039	0.845	
**ss**	3.20	1.11	1.60	0.81	2.83	0.65	1.00	0.55	3.17	0.79		1.67	0.76	1.83	0.87	0.83	0.31	–	–	
**wl**	4.00	1.48	5.20	1.74	5.33	1.15	3.60	1.17	5.67	1.63		3.83	1.08	3.50	0.99	2.33	1.17	0.939	0.339	
**re**	2.20	1.96	1.80	1.20	0.67	0.67	0.40	0.24	0.00	0.00		0.33	0.33	0.83	0.40	0.00	0.00	–	–	
**ju**	0.00	0.00	0.40	0.40	0.00	0.00	0.00	0.00	0.00	0.00		0.00	0.00	0.00	0.00	0.00	0.00	–	–	
**fbg**	1.00	0.77	1.80	0.58	4.17	0.95	4.40	1.29	2.83	0.87		2.67	0.99	2.83	0.65	0.50	0.34	–	–	
**hbg**	0.00	0.00	0.40	0.24	3.17	0.91	1.00	1.00	0.33	0.33		0.33	0.33	1.33	0.49	0.00	0.00	–	–	
**im**	0.00	0.00	6.60	2.69	1.50	0.62	7.00	2.72	2.00	1.29		6.33	2.01	2.50	0.89	11.00	1.97	–	–	
**DURATIONS**																				
	**HDC**^**+/+**^										**HDC**^**−/−**^									
	**NS-VEH**		**S-VEH**		**NS-OEA**		**S-OEA**		**NS-VEH**			**S-VEH**		**NS-OEA**		**S-OEA**				
**Behavior**	**Mean (n)**	**SE**	**Mean**	**SE**	**Mean**	**SE**	**Mean**	**SE**	**Mean (s)**	**SE**		**Mean**	**SE**	**Mean**	**SE**	**Mean**	**SE**	**F**		**p**
**cs**	60.41	4.23	15.86	5.19	35.53	2.63	30.40	9.61	25.25	1.71		20.09	4.69	36.27	6.18	12.53	4.73	**15.747**		**<0.001**
**cl**	11.84	4.44	2.76	0.80	14.64	2.23	4.80	0.64	3.30	2.58		3.48	1.96	9.79	2.34	2.52	0.00	–		–
**cc**	6.28	1.83	0.00	0.00	5.78	5.22	0.00	0.00	0.00	0.00		0.00	0.00	28.70	14.50	0.00	0.00	–		–
**cr**	2.34	1.46	1.41	0.21	0.60	0.18	2.76	0.62	3.28	0.64		3.03	1.05	0.92	0.39	1.65	0.70	1.002		0.325
**wa**	38.52	4.06	26.50	2.46	30.15	2.12	26.63	6.40	34.28	5.44		26.14	3.90	18.50	3.42	21.15	5.53	0.033		0.856
**ps**	20.87	2.51	69.34	10.41	41.47	5.59	57.20	12.85	65.83	6.69		69.62	7.49	56.11	11.08	71.11	8.14	3.273		0.079
**ss**	2.57	0.92	3.09	0.29	1.72	0.52	1.11	0.45	3.04	0.67		1.83	0.42	1.47	0.28	1.51	0.33	–		–
**wl**	9.96	3.08	9.24	4.33	8.38	0.91	5.76	1.15	8.46	2.74		6.75	2.00	5.13	1.21	4.37	2.24	0.161		0.691
**re**	4.74	4.38	3.56	1.52	5.24	0.00	0.58	0.42	0.00	0.00		0.92	0.00	2.01	0.70	0.00	0.00	–		–
**ju**	0.00	0.00	2.16	0.00	0.00	0.00	0.00	0.00	0.00	0.00		0.00	0.00	0.00	0.00	0.00	0.00	–		–
**fbg**	4.24	1.80	5.66	1.43	8.53	0.96	6.84	2.53	7.26	1.17		6.07	1.59	5.22	0.55	5.82	0.18	–		–
**hbg**	0.00	0.00	1.16	0.40	6.50	2.05	6.28	0.00	3.08	0.00		1.16	0.00	3.34	1.30	0.00	0.00	–		–
**im**	0.00	0.00	27.80	2.31	5.47	2.02	23.52	7.93	5.32	1.90		19.85	7.27	7.82	2.72	36.52	8.15	–		–

Here the results are presented as T-pattern strings, which are the textual description of a pattern and its hierarchical composition. Occurrences and durations are presented as mean number ± SE performed by each subject during the testing time. Differences among groups were assessed using three-way ANOVA followed by Bonferroni's post-hoc test for multiple comparisons. Theories, concepts and procedures concerning the detection and the analysis of T-patterns can be found in several citations (Casarrubea and Di Giovanni, 2020; Casarrubea et al., 2018; Magnusson, 2000, 2020).

### 2.6 Novel object recognition test

This paradigm was performed as previously reported (Provensi et al., 2016). Briefly, the novel object recognition test consisted of a 10 min habituation in the empty arena; a 5 min training session (T1) during which mice were placed in the test arena containing two identical plastic objects; test session (T2), during which each mouse was again placed in the test arena for 5 min in the presence of one familiar object and a novel one. The time spent exploring either object during T1 and T2 was recorded by an experienced observer unaware of the treatments. The position of the objects (left/right) was randomized to prevent biases. Exploration was defined as sniffing or touching the objects with the nose and/or forepaws. Sitting on or turning around the objects was not considered exploratory behavior. T1 was performed 24 h after Habituation, whereas T2 was carried out 1 h after T1 to evaluate short term memory**.** To analyze recognition memory, a discrimination index was calculated as the ratio of the amount of time spent exploring the novel object over the total time spent exploring the familiar and novel objects in the retention trial.

### 2.7 Oxytocin immunostaining protocol

Mice were deeply anesthetized with pentobarbital sodium (80 mg/kg ip) and transcardially perfused with ice-cold sodium phosphate buffer (0.1 M PBS, pH 7.4) followed by fixative solution containing 4% paraformaldehyde. Fixed brains were removed from the skull, postfixed overnight, and then cryoprotected in 20% sucrose-phosphate buffer (48 h at 4 °C) stored at −20 °C until processed for immunostaining. Three series of 30 μm of coronal brain sections containing the PVN were cut on a cryostat (model HM550; Thermo Fisher Scientific, Kalamazoo, MI, USA) and stored in a solution of phosphate buffer (PB 0.1 M pH 7.4) and NaN3 (0.02 M) at +4 °C until stained. Oxytocin immunostaining was performed according to our previous studies (1–3). Briefly, a first series of 30 μm-thick free-floating coronal sections containing the PVN and SON were rinsed with PB (0.1 M pH 7.4) and then incubated for 2h in a blocking solution containing 3% BSA (bovine serum albumin, SERVA), 0,3% Triton X-100, 0.3 M glycine and 4% of NGS (normal goat serum, Invitrogen). After additional washes, sections were incubated overnight at 4 °C with the primary antibody (mouse anti-OXY monoclonal primary antibody 1:1000 dilution, MAB 5296, Millipore) diluted in the blocking solution. Sections were then rinsed in PB containing 0,3% Triton X-100 and incubated with the secondary antibody Alexa Fluor 488 (1:400 dilution; Invitrogene) for 2h. After final washes, slides were cover-slipped with Fluoromount (Sigma Aldrich). The specificity of the immunostaining was assessed by the absence of signal in control brain sections containing the regions of interest that underwent the same protocol procedure except for the incubation with the primary antibody.

### 2.8 Brain section analysis

Analysis was performed according to (Romano et al., 2017). Brain sections obtained were observed under a Nikon Eclipse 80i microscope equipped with a color charge-coupled device camera and controlled by the software NIS-Elements-BR (Nikon). Slices were photographed under epifluorescent conditions. The mouse brain atlas by Paxinos and Franklin (2007) was used as reference for the localization of the brain areas of interest. Analysis of oxytocin positive cells was conducted manually by counting each OXY-positive cell within the PVN area (number of oxytocin cells/mm^2^). The investigator was blind to animal treatment; measurements were obtained in at least three consecutive tissues sections per animal containing the desired structure.

### 2.9 Statistics

Data were analyzed by using a three-way ANOVA and Bonferroni's post hoc test for multiple comparisons, by using Prism 9.0.0 (Graphpad Software, La Jolla, CA). Data are presented as mean ± SEM and statistical significance was set at p < 0.05.

## 3 Results

Mice of both HDC^+/+^ and HDC^−/-^ genotypes were subjected to the protocol shown in Fig. 1. Non-stressed mice of either genotype were left undisturbed in their home cage until sacrifice.

### 3.1 Effect of stress and OEA on bodyweight change and food consumption

**Fig. 2:**
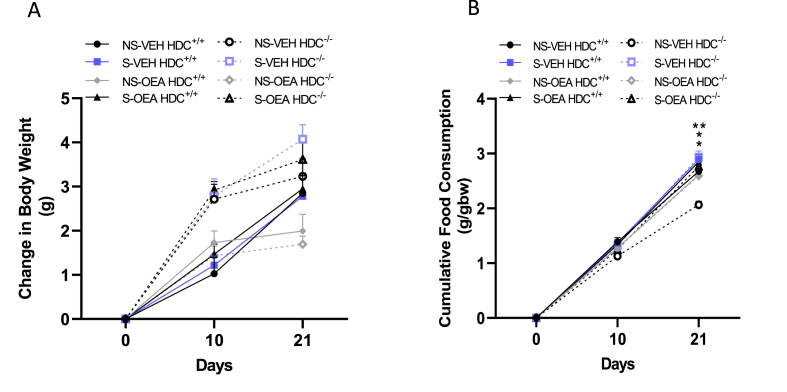
Effects of stress and OEA (10 mg/kg) on weight gain (A) and cumulative food consumption (B) at T0 before initiating stress protocol, at T10 before starting OEA treatment, at T21 on completion of the stress procedure. Data are shown as means ± s.e.m. of 6–9 mice/experimental group. **P < 0.01, *P < 0.05, within genotypes NS, non-stressed; S, stressed; VEH, vehicle; OEA, oleoylethanolamide (10 mg/kg i.p.).

Mice of both genotypes gained comparable weight as three-way ANOVA showed a significant effect of genotype_x_time and treatment independently of stress (F_genotype x time (1.895, 41.30)_ = 106.8, P<0.0001; F_treatment (1,23)_ = 11.73; P<0.01; F_stress (1,23)_ = 3.968, P=0.0584; F_interaction(5,109)_ = 2.070, P=0.0747; Fig. 2A). Bonferroni's post-hoc test though, failed to show significant differences between groups on different days of treatment regardless of genotype or stress. Regarding food consumption, three-way ANOVA showed a significant interaction between genotype, stress and treatment over time (F_genotype x time (2.044, 44.,57)_ = 2688; P < 0.0001; F_treatment (1,23)_ = 22.52, P < 0.0001; F_stress (1,23)_ = 0.1770, P = 0.6779; F_interaction(5,109)_ = 9.329, P < 0.0001; Fig. 2B). Bonferroni's post hoc analysis revealed that non-stressed HDC^−/-^ mice treated with vehicle ate significantly less than non-stressed and stressed mice treated with OEA and stressed mice treated with vehicle of both genotypes.

The absence of the well-documented anorexigenic effects of OEA (Fu et al., 2003, 2005; Romano et al., 2015) in our study is attributable to the experimental design. When administered just before the onset of the dark phase, during which rodents are active and consume food, OEA affects feeding behavior by producing an anorexigenic effect observable both in acute (Provensi et al., 2014) or chronic protocols (Fu et al., 2003); (Di Paola et al., 2018). However, the assessment of the satiety effect of OEA was beyond the aim of the present study; in our experimental conditions OEA treatment does not affect eating-behavior because the time of administration is too far from the consummatory phase of the animals and because of the short half-life of the drug.

### 3.2 Effect of OEA and histamine deprivation in mice social interaction

**Fig. 3:**
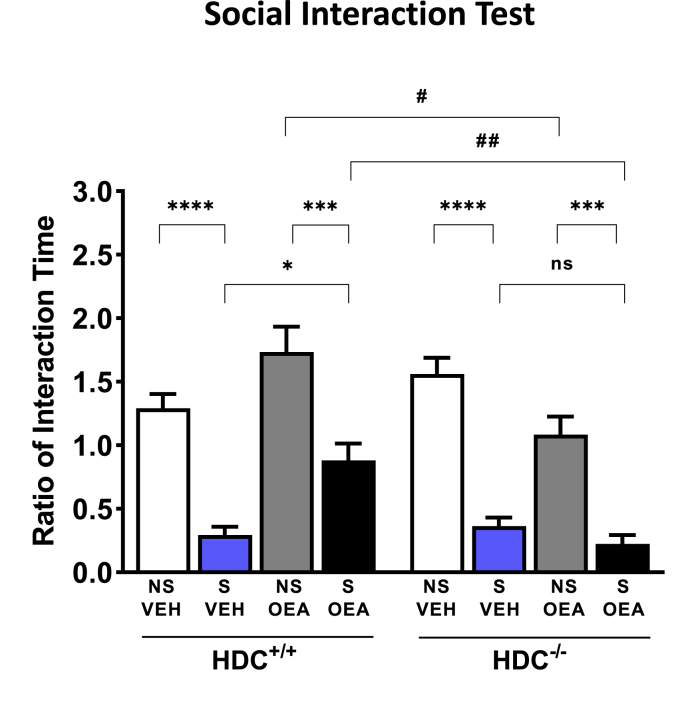
Effect of OEA administration on social-avoidance behavior induced by stress. Repeated social defeat stress induced social avoidance of both HDC^+/+^ and HDC^−/-^ mice expressed as the ratio between the time a mouse spent in the interaction zone in the presence and in the absence of a target CD-1. Data are presented as means ± s.e.m. of 6–9 mice/experimental group ****P < 0.0001, ***P < 0.001, *P < 0.05, within genotypes; ^##^P < 0.01; ^#^P < 0.05, between genotypes. NS, non-stressed; S, stressed; VEH, vehicle; OEA, oleoylethanolamide (10 mg/kg i.p.).

The genetic lack of histamine did not affect sociability, as non-stressed mice of either genotypes and treatment interacted with the caged CD1 mouse, as shown in Fig. 3. Three-way ANOVA revealed that CSDS and OEA affected the behavior of both HDC^+/+^ and HDC^−/−^ mice (F_stress(1,46)_=125.9, P<0.0001 (F_treatment (1,46)_=1.917; P=0.1729; F_genotype(1,46)_= 7.696; P< 0.01; F_interaction(1,46)_=22.35, P<0.0001). Bonferroni's post hoc test showed that the total time vehicle-treated, stressed mice of both genotypes spent in the proximity of the caged CD1 mouse was significantly shorter than that of non-stressed mice. OEA effect on physiological conditions was different between genotypes, as non-stressed HDC^+/+^ mice treated with OEA spent more time in the proximity of the caged mouse with respect to null mice. This result is not surprising as oxytocin promotes socialization (Witt et al., 1990) and presumably requires the histaminergic neurotransmission to fully unfold its effect (see also Fig. 6). OEA though, partially prevented the social aversion of stressed HDC^+/+^ but did not change the behavior of stressed HDC^−/−^ mice (Fig. 3).

### 3.3 Effect of OEA and histamine deprivation in mice behavioral patterns

**Fig. 4:**
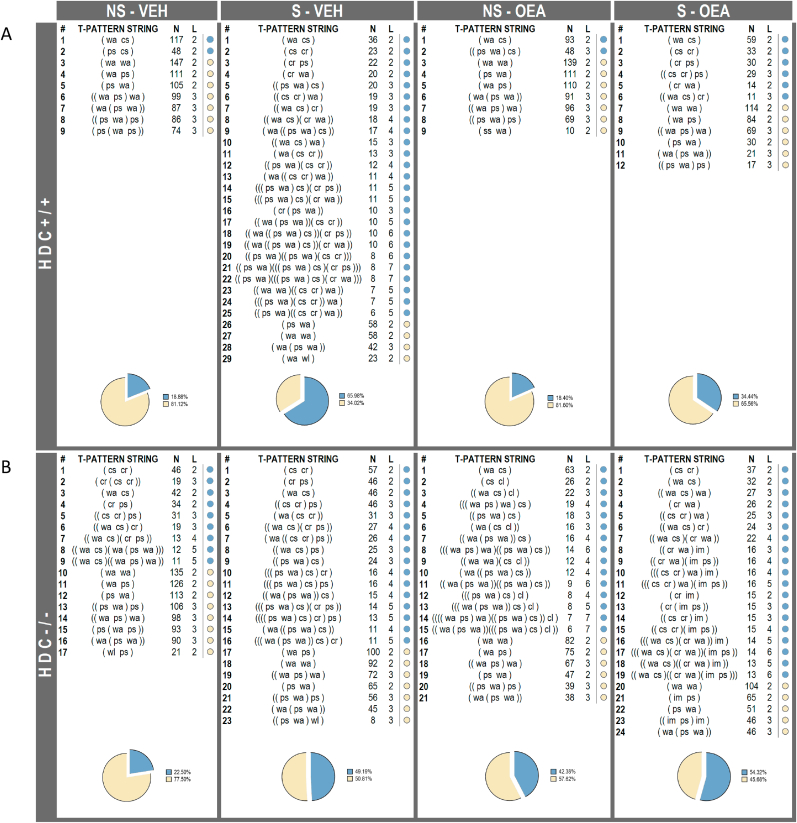
T-pattern analysis for each of the study group during the Social Interaction test. A) Effect of stress and OEA on T-patterns of HDC^+/+^ mice. B) Effect of stress and OEA on T-patterns of HDC^−/-^ mice. T-pattern string = textual representation of each pattern of different composition; brackets indicate the hierarchical structure. N = overall occurrences of each pattern of different composition. L = number of behavioral events within each T-pattern string. Pie charts represent percent distribution of T-patterns containing *Cage Related* (blue) and *Other Events* (yellow). See Ethogram in Table 1 for abbreviations. NS, non-stressed; S, stressed; VEH, vehicle; OEA, oleoylethanolamide (10 mg/kg i.p.). (For interpretation of the references to color in this figure legend, the reader is referred to the Web version of this article.)

Stress and OEA did not affect the locomotion of mice of either genotype as the time spent in the central or peripheral zone of an empty arena was not affected by stress or treatment (F_interaction Center (1,21)_=0.04375, P=0.8363; F_interaction Pheriphery (1,21)_ = 2.314, P=0.1431; Supplementary figure 1**).** Despite the gross similarity of behaviors between non-stressed HDC^+/+^ and HDC^−/-^ mice, we know that brain histamine contributes to the qualitative features of displayed motor behaviors not only in animals (Santangelo et al., 2017), but also in humans (Baldan et al., 2014). Therefore, we analyzed the complex behavioral sequence of experimental mice during the social interaction test. A preliminary quantitative analysis was based on an ethogram that encompassed *Cage related* (i.e., in the proximity of the cage holding the CD1 mouse) and *Non cage related* (*Other) events* (i.e., displayed in the arena; Table 1). Mean (±s.e.m.) occurrences and duration of each specific component of the ethogram are shown in Table 2. The statistical analysis was possible only for the sufficiently represented behaviors. Cage sniffing was by far the most frequent and long-lasting behavior among *Cage Related* components and was performed by both HDC^+/+^ and HDC^−/-^ mice. ANOVA showed statistically significant differences in mean *cage sniffing (cs)* occurrence [F_genotype__(1, 37)_=5.287 P<0.05; F_treatment__(1, 37)_ = 20.37, P<0.0001; F_interaction__(1, 37)_ = 9.650 P<0.01] and mean duration [F_genotype (1, 37)_=10.81, P<0.01; F_treatment(1, 37)_=28.91, P<0.0001; F_interaction__(1, 37)_ = 15.75, P<0.001] among the different experimental groups. Stressed HDC^+/+^ mice treated with vehicle sniffed the cage less frequently and for a much shorter time than non-stressed congeners (P < 0.01; P < 0.0001, respectively). OEA did not significantly affect these parameters in non-stressed and stressed mice. On the other hand, chronic stress, despite decreasing significantly the time of social interaction (Fig. 3B), did not impact on *cs* occurrence and duration of HDC^−/−^ mice. Of note, *cs* duration of non-stressed HDC^−/−^ mice was much shorter than that of HDC^+/+^ mice (P < 0.001). OEA decreased *cs* duration of stressed HDC^−/−^ mice compared to non-stressed HDC^−/−^ congeners (P <0.05). Walking *(wa)* and Place Sniffing (*ps*) were the most frequent *Other events* of both genotypes. The mean *wa* and *ps* occurrence and duration of HDC^+/+^ and HDC^−/−^ mice did not change significantly with any of the treatments. The striking differences between the behaviors of the two genotypes are better appreciable in the T-pattern analysis. The T-pattern analysis is conceived to detect events in time-ordered sequences characterized by statistically significant constraints among them (Casarrubea et al., 2015); (Santangelo et al., 2017). Mice behavioral structure was characterized by a complex temporal organization in the arena during the social interaction. Nine T-pattern strings were detected in non-stressed HDC^+/+^ mice treated with either vehicle or OEA, encompassing 2 or 3 events in their structure, with only 2 of them containing *Cage Related* events, (Fig. 4A, see Table 1 for abbreviations). The stressed group showed a strikingly more complex behavioral structure, as HDC^+/+^ mice performed a total of 29 T-pattern strings, 25 of which containing C*age Related Events*, mostly Cage Sniffing followed by Cage Retraction (cr). The *Other Events* comprised mainly Walking and Place Sniffing (yellow dots). Stressed HDC^+/+^ mice treated with OEA performed 12 T-pattern strings very similar in structure to non-stressed mice, although *Cage Related Events* within each string were more numerous (6 out of 12, exclusively Cage Sniffing and Cage Retraction).

Non-stressed HDC^−/-^ mice showed a strikingly more complex behavioral structure compared to non-stressed HDC^+/+^ mice (Fig. 4B), similar to that observed in CD1 mice pharmacologically deprived of histamine (Santangelo et al., 2017). Non-stressed HDC^−/−^ mice treated with vehicle or OEA displayed 17 and 21 T-pattern strings, respectively, with up to 7 events in the structure (NS-OEA). Nine and 15 T-pattern strings contained *Cage Related* events in NS-Veh and NS-OEA mice, respectively (Fig. 4B; blue dots)**.** Stress increased considerably the number of complex patterns of HDC^−/-^ mice, in particular T-patterns containing *Cage Related Events* (16 out of 23). OEA did not significantly change the total number of T-pattern strings of stressed mice, rather it further increased the number of patterns containing *Cage Related Events* (19 out of 24, blu dots). The pie charts in Fig. 4 represent the percentage of *Cage Related* (blue) and *Other* T-patterns (yellow) displayed by each experimental group.

**Fig. 5:**
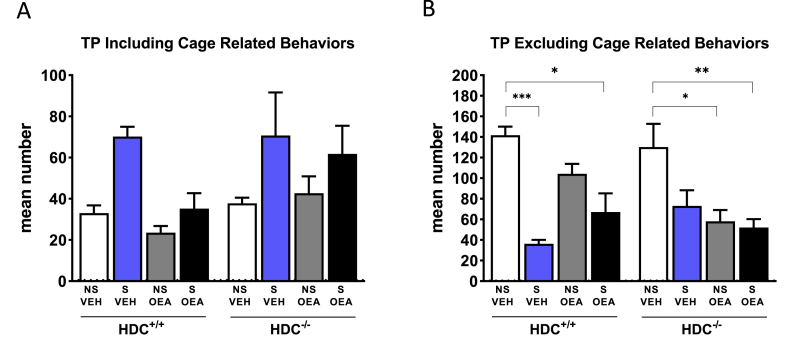
T-pattern graphs representing patterns including A) or excluding B) Cage Related Events in their composition. Results are presented as mean number ±s.e.m of T-pattern strings of the behavior of each experimental group during the social interaction test. N = 5–6 mice/experimental group. ***P < 0.001, **P < 0.01, *P < 0.05. NS, non-stressed; S, stressed; VEH, vehicle; OEA, oleoylethanolamide (10 mg/kg i.p.).

Fig. 5 shows the mean number of T-patterns including or excluding cage related events performed by each mouse. When considering TP including cage related events three-way ANOVA showed significant difference among groups with regard to stress conditions (F_stress(1,37)_ = 11.31, P < 0.01). However, no significant differences among experimental groups were found with Bonferroni's post hoc test. TP that do not include cage related events (Fig. 5B) were considerably different among experimental groups (F_stress (1,37)_ = 28.20; P < 0.0001; F_treatment (1, 37)_ = 9.481, P < 0.01; F_genotype x stress (1, 37)_ = 4.205, P < 0.05; F_stress x treatments (1, 37)_ = 6.627, P < 0.05; F_interaction (1, 37)_ = 4.976, P < 0.05). HDC^+/+^ stressed mice performed significantly less T-patterns that non stressed controls treated with vehicle or OEA. OEA partially prevented the effect of stress. Of note, OEA did not affect the behavioral patterns of HDC^+/+^, but it decreased the mean number of non-cage related T-patterns of both non-stressed and stressed HDC^−/-^ mice (Fig. 5B).

**Fig. 6:**
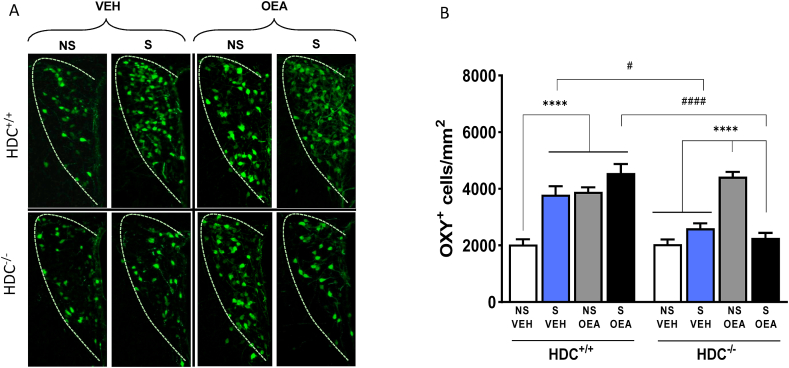
Effect of stress and OEA on oxytocin immunofluorescence in the PVN. A) Representative images showing oxytocin immunopositive cell in the PVN of HDC^+/+^ and HDC^−/-^ mice. (B) The bar graphs represent the quantitative data shown in A. Results are expressed as means ± s.e.m. N = 4-6 slices/brain of 3 mice/group; ****P < 0.0001, within genotypes; ^####^P< 0.0001; ^#^P< 0.05 between genotypes. NS, non-stressed mice; S, Stressed mice; VEH, vehicle-treated mice; OEA, oleoylethanolamide (10 mg/kg i.p.).

### 3.4 Effect of stress and OEA on oxytocin immunostaining

Three-way ANOVA revealed a significant interaction among the three factors: genotype, stress condition and treatment F_stress (1,218)_ =31.46, P<0.0001; F_treatment (1,__218)_=1.475, P=0.2258; F_genotype (1,218)_=18.57, P<0.0001; F_interaction__(1,218)_=0.7345, P=0.3924). Bonferroni's post hoc test showed that OEA effect on physiological conditions was similar between genotypes, as non-stressed HDC^+/+^ and null mice treated with OEA showed higher oxytocin immunoreactivity with respect to their vehicle treated controls (Fig. 6). Chronic social stress increased oxytocin immunofluorescence in the PVN of HDC^+/+^ mice and OEA further increased oxytocin signal. Chronic stress did not affect oxytocin immunofluorescence in the PVN of HDC^−/−^ mice, nor did OEA, thus supporting the hypothesis that the beneficial effects of OEA by means of oxytocin presumably requires the histaminergic neurotransmission.

### 3.5 Effects of OEA on CSDS-induced short term memory impairment

**Fig. 7:**
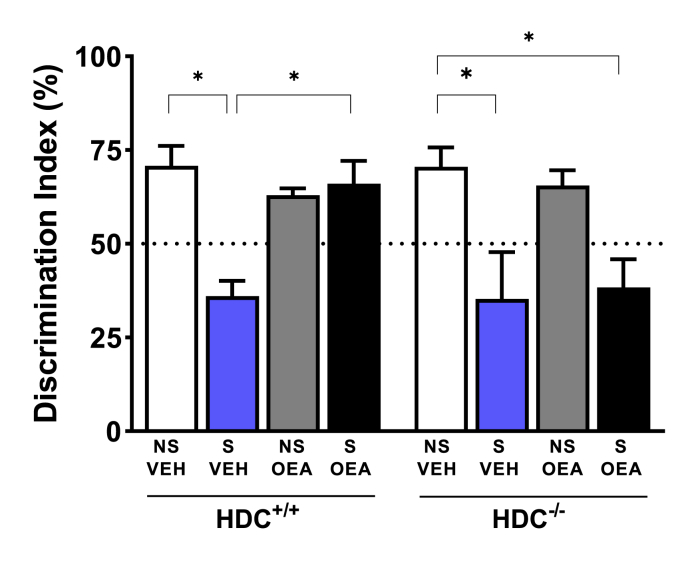
Effect of OEA administration on stress-induced cognitive impairment in the novel object recognition test. Social defeat stress affected the performance of both HDC^+/+^ and HDC^−/-^ mice when tested 1 h after training, as indicated by the discrimination index. Results are expressed as means ± s.e.m. 6–8/experimental group. *P < 0.05. NS, non-stressed mice; S, Stressed mice; VEH, vehicle-treated mice; OEA, oleoylethanolamide (10 mg/kg i.p.).

Fig. 7 shows the performance of mice in the novel object recognition test. The discrimination index were significantly different among experimental groups (F_stress (1,43)_ = 25.24, P < 0.0001; F_treatment (1, 43)_ = 6.015, P < 0.05; F_genotype (1,43)_ = 1.927, P = 0.1722; F_interaction__(1,43)_ = 1.661, P = 0.2044). When tested 1 h after training, three weeks of CSDS had a negative effect on mice ability to discriminate between the familiar and new object, which indicates that stressed mice of both genotypes had a cognitive impairment. OEA treatment rescued the behavioral impairment of HDC^+/+^ mice only, as stressed, OEA-treated HDC^−/-^ mice did not show any memory improvement.

## 4 Discussion

Chronic uncontrollable stress is a major risk factor for the development of metabolic and psychiatric disorders. A useful preclinical model to understand the molecular mechanisms underlying affective-like disorders is the social defeat stress which results in the development of depressive-like behavioral impairments characterized by enduring deficits in metabolic processes (van der Kooij et al., 2018), inflammation **(**Patki et al., 2013**)**, social interactions (Golden et al., 2011) and memory (Monleón et al., 2016).

The main finding of the present study was that repeated daily treatment with OEA prevented social interaction deficits and short-term memory impairment induced by chronic social stress and that this effect required the integrity of the histaminergic system. Other neurotransmitters’ systems such as the noradrenergic (Finnell et al., 2019) and orexinergic (Eacret et al., 2019; Grafe et al., 2018) are known to contribute to social stress in different ways. Our study uncovers previously unidentified neural signaling pathways involved in stress responses.

It is well documented that chronic stress reduces social motivation and social interactions (reviewed in (Sandi and Haller, 2015), therefore it is not surprising that mice of either genotype reduced dramatically the interaction time with the cage holding the aggressive mouse. Though, the preventive effect of OEA was unexpected, furthermore OEA was effective only in wild type, but not in HDC^−/-^ mice. A deeper level of analysis of behavioral dynamics is afforded by the use of TPA, an approach widely used to study also patients affected by movement and behavioral disorders (Aiello et al., 2020). The behavioral patterning elicited by the social interaction revealed a more complex picture than predicted by the plain quantitative evaluations of social interaction. We observed several differences between non-stressed HDC^+/+^ and HDC^−/-^ mice and differences in the impact of stress and OEA on the two genotypes. First of all, non-stressed HDC^−/-^ mice showed a marked increase in pattern complexity (i.e., the number of T-pattern of different composition, see Fig. 4) with respect to HDC^+/+^ mice. The absence of histamine in the brain profoundly affected how single components of the T-pattern strings interacted in time, contributing to the configuration of repetitive behavioral sequences. A similar change in complexity and number of T-patterns was observed also in CD1 mice pharmacologically deprived of histamine with i.c.v. injections of α-fluoromethylhistidine, a suicide inhibitor of histidine decarboxylase (Santangelo et al., 2017).

When exposed to chronic social stress, HDC^+/+^ mice sniffed the cage containing the aggressive mouse far less often and for shorter bouts (*cs* in Table 2). Furthermore, stressed HDC^+/+^ mice displayed increased complexity and number of T-pattern strings encompassing *Cage Related Events*. We interpret such an aspect as indicative of a conflict between an engrained approaching behavior and fear caused by the presence of the aggressive mouse. In other words, HDC^+/+^ mice displayed more varied T-pattern strings in their composition and containing a higher number of *Cage Related Events* suggesting a remarkable reorganization of the anxiety-related behavior and, more in general, an evident reorganization of the behavioral patterning. A similar reorganization of anxiety related behaviors was also observed after chronic administration of low doses of nicotine in rats (Casarrubea et al., 2020). OEA partially restored the behavioral sequences, similar to that displayed by non-stressed mice, in terms of T-pattern string complexity and mean number of T-patterns (both *Cage Related and Other Events)*. In our paradigm, OEA seems to alleviate an anxiety-like behavior induced by repeated social stress in mice. Interestingly, OEA decreased stressed induced binge-eating in female rats (Romano et al., 2020), supporting the pharmacological potential of OEA for the treatment of stress-related disorders.

The ratio of interaction time of stressed HDC^−/-^ mice was not dissimilar from that of stressed HDC^+/+^ mice (Fig. 3), although T-pattern strings containing *Cage Related Events* were less numerous (16 vs 25), as well as less complex (up to 5 in HDC^−/-^ and up to 7 components in HDC^+/+^ mice, Fig. 4). Furthermore, the mean occurrence and duration of each component was not statistically different among stressed and non-stressed HDC^−/-^ mice regardless of the pharmacological treatment.

Apparently HDC^−/-^ mice do remember the encounters with the aggressive CD1 mouse, as shown by the social interaction index, but the repertoire and duration of their behaviors, along with the T-pattern strings are markedly different from those of HDC^+/+^ mice. It has been reported that HDC^−/-^ mice reproduce a mutation associated with Tourette's syndrome (Baldan et al., 2014; Pittenger, 2020). We previously suggested that histamine deficiency, although not associated with the manifestation of stereotypies, may represent a predisposing behavioral phenotype underlying the enhanced stereotypical tic-like behaviors of Tourette's syndrome (Santangelo et al., 2017). A possible explanation for the overall behavior of HDC^−/-^ mice is the incapacity of elaborating appropriate patterns which are unlocked from their stereotypical repertoire.

Stressed HDC^−/-^ mice treated with OEA showed no significant differences from vehicle-treated, stressed mice. OEA did not modulate the behavioral repertoire of histamine deficient mice. HDC^−/-^ mice apparently have a dysregulated striatal and prefrontal cortex function (Rapanelli et al., 2017a); (Rapanelli et al., 2017b); (Santangelo et al., 2017) that may contribute to the aberrant behaviors and memory impairment of these mice and the lack of response to OEA. Indeed, CSDS also compromised the short-term memory of both HDC^+/+^ and HDC^−/-^ mice, and the procognitive effects of OEA (Campolongo et al., 2009) were lost in histamine-deficient mice.

The neurophysiological mechanisms underlying OEA and brain histamine cross talk are not known. OEA and the histaminergic system presumably do not interact directly in the brain. When examining certain histaminergic brain projection areas, we found that OEA induced c-Fos expression in HDC^+/+^, but not in HDC^−/-^ mice (Umehara et al., 2016); however, OEA increased c-Fos expression in the nucleus of solitary tract (NST), the main brainstem nucleus activated by vagal afferents relaying OEA signaling from the periphery (Rodríguez de Fonseca et al., 2001), in the CNS of both genotypes. OEA-induced activation of the NST therefore, precedes the stimulation of histaminergic neurons, suggesting that in HDC^−/-^ mice the signaling relayed to the NST is interrupted further down the line. Hence, the absence of histamine appears to be responsible for the inefficacy of OEA on the observed behavioral parameters.

Oxytocin is known for promoting socialization interaction and may also induce avoidance of potentially unfavorable social contexts (Neumann and Slattery, 2016; Steinman et al., 2019); we therefore, examined oxytocin immunofluorescence in the PVN of our experimental animals. OEA augmented oxytocin positive neurons in the PVN of non-stressed mice of both genotypes, which is in agreement with our previous observation (Provensi et al., 2014). Chronic social defeat stress increased the number of oxytocin immunofluorescent neurons in the PVN of HDC^+/+^ mice, but not in the PVN of HDC^−/-^ mice, which is surprising and merits further investigation. OEA further increased the immunofluorescence of stressed HDC^+/+^ mice, but failed to affect HDC^−/-^ mice. It therefore appears that in a disturbed situation as following chronic social defeat, OEA and presumably oxytocin do not exert their beneficial effect if the histaminergic system is impaired. We cannot easily offer a valid model based on oxytocin expression to explain the complex behavioral outcome of our protocols. Steinman et al. (2019) proposed that distinct oxytocic brain circuits mediate social avoidance or social approach, enhancing the relevance of both positive and negative social interactions. It would be interesting to know if the activated neurons of HDC^+/+^ mice project to different brain nuclei that subserve different functions and that require histaminergic input. In this regard, histaminergic axons originating from the TMN in the posterior hypothalamus to innervate almost all central nervous system areas, a feature consistent with a function over a host of physiological processes, including the regulation of the sleep-wake cycle, appetite, endocrine homeostasis, cognition and emotion. There is much experimental evidence demonstrating that histaminergic neurons are heterogeneous, organized into functionally-distinct circuits impinging on different brain area, and displaying selective control mechanisms (Munari et al., 2013). This suggests independent functions of subsets of histamine neurons according to their respective origin and terminal projections (Blandina et al., 2012)

## 5 Conclusions

In conclusion, the beneficial effects of OEA occur only in the presence of a functioning histaminergic system. This is reflected not only in the social interaction test and the novel object recognition test, but also in the ethological study. The T-pattern analysis by describing fine behavioral features and differences between groups, suggests a fundamental role of histamine on the organization of repetitive behavioral sequences and revealed that the behavioral re-organization induced by OEA does not occur in the absence of histamine. These data substantiate our hypothesis of a *permissive* role of brain histamine on the behavioral effects of OEA. In this regard, it is notable that histamine receptor ligands are among the most used drugs worldwide; hence, understanding the impact of histamine and these compounds on stress consequences may help improve their pharmacological profile and unravel unexplored therapeutic applications.

## Funding

This research was supported by ERA-HDHL Project AMBROSIAC and Fondazione Ente Cassa di Risparmio Firenze to MBP.

## CRediT authorship contribution statement

**Barbara Rani:** Methodology, Validation, Formal analysis, Investigation. **Andrea Santangelo:** Methodology, Software, Formal analysis, Data curation. **Adele Romano:** Conceptualization, Methodology, Validation. **Justyna Barbara Koczwara:** Methodology, Formal analysis. **Marzia Friuli:** Methodology, Formal analysis. **Gustavo Provensi:** Methodology, Formal analysis. **Patrizio Blandina:** Conceptualization, Writing – review & editing. **Maurizio Casarrubea:** Software, Methodology, Writing – original draft, preparation. **Silvana Gaetani:** Conceptualization, Formal analysis. **Maria Beatrice Passani:** Conceptualization, Formal analysis, Writing – review & editing, Supervision, Funding acquisition. **Alessia Costa:** Conceptualization, Methodology, Investigation, Formal analysis, Writing – original draft.

## Declaration of competing interest

The authors declare no conflict of interest.
