# Supplementary figures and images for: Brain histamine and oleoylethanolamide restore behavioral deficits induced by chronic social defeat stress in mice
Source: Neurobiol Stress. 2021 Mar 17;14:100317. doi: 10.1016/j.ynstr.2021.100317 (PMC8039856; doi:10.1016/j.ynstr.2021.100317)

## Slide 1
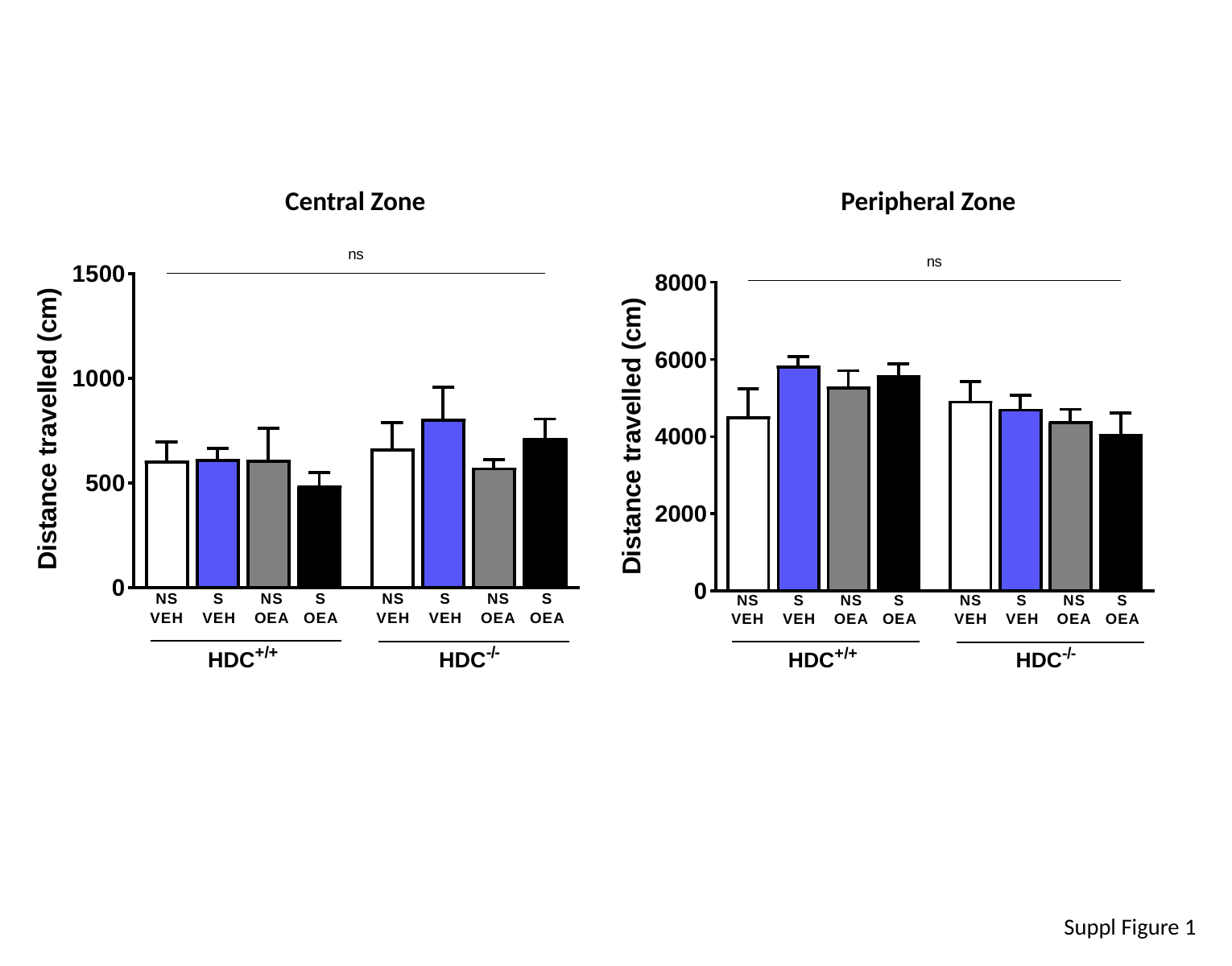

Central Zone
Peripheral Zone
Suppl Figure 1

Supplement: Multimedia component 1 [file mmc1.pptx]
